# Survival of Chinese people with type 2 diabetes and diabetic kidney disease: a cohort of 12 -year follow-up

**DOI:** 10.1186/s12889-019-7859-x

**Published:** 2019-11-09

**Authors:** Zihou Zhao, Lili Huo, Lianying Wang, Lijuan Wang, Zuodi Fu, Yufeng Li, Xiaohong Wu

**Affiliations:** 1 First Clinical Medical College, Nanjing Medical University, Nanjing, 210029 People’s Republic of China; 2 Department of Endocrinology, Beijing Jishuitan Hospital, No. 31, Xinjiekou East Street, Xicheng District, Beijing, 100035 People’s Republic of China; 3 Department of Endocrinology, Capital University Beijing Friendship Hospital Pinggu Campus, No.59 Xinping North Road, Pinggu District, Beijing, 101200 People’s Republic of China; 4 Department of Endocrinology, First Affiliated Hospital of Nanjing Medical University, No. 300 Guangzhou Road, Nanjing, 210029 People’s Republic of China

**Keywords:** Diabetic kidney disease, Mortality, Survival analysis, Type 2 diabetes

## Abstract

**Background:**

The prevalence of type 2 diabetes has grown significantly in China. However, little is known about the survival outcome of people with type 2 diabetes and diabetic kidney disease (DKD). The purpose of this study is to examine the survival of this population and the risk factors for mortality in one suburb cohort of Beijing, China.

**Methods:**

Four hundred and forty-five people with DKD (48.8% male, age at onset of diabetes 48.8 ± 11.0 years, age at enrollment 57.5 ± 11.6 years) were enrolled in one suburb of Beijing, China between January 1st, 2003 and December 31st, 2015. Mortality ascertainment was censored by December 31st, 2015. Survival analysis was performed by Kaplan–Meier analysis, and Cox proportional hazards regression models were served for risk factor analysis of mortality. The Chiang method was used to estimate life expectancy by age.

**Results:**

A total of 78 deaths were identified during the 3232 person-years of follow-up. Multivariate Cox regression analysis showed significantly higher risks of mortality with respect to older age, higher systolic blood pressure (SBP), lower body mass index (BMI) and lower estimated glomerular filtration rate (eGFR). The life expectancy at age of 50 was estimated to be 12.3 (95%, CI: 9.0–16.1) years. Circulatory disease was the leading cause of death in this population (accounting for 43.6% of all deaths), followed by diabetic complications (33.3%) and respiratory disease (6.4%).

**Conclusions:**

Data from one Chinese cohort from 2003 through 2015 showed that people with DKD faced higher risk of death and shorter life expectancy. Factors significantly increasing risk of death included older age, higher SBP, lower BMI and lower eGFR. There is an urgent need to early detection, closely monitoring and effective intervention on DKD.

## Background

The prevalence of type 2 diabetes is increasing rapidly worldwide. The epidemic of diabetes has had a marked impact on the development of diabetic kidney disease (DKD) [1]. DKD is defined as diabetes with kidney involvement, presenting as albuminuria and/or an impaired glomerular filtration rate (GFR). A study from China using a general population-based, nationally representative sample of 47,204 participants from 2009 through 2010 revealed that approximately 21.3% of the people with diabetes were classified as having DKD, and 60% of the people with DKD have preserved kidney function with slightly increased albuminuria [2]. Albuminuria and GFR are independently and additively correlated with increased risks of cardiovascular disease (CVD) events, CVD mortality, and total mortality among people with diabetes [3]. Standardized ten-year cumulative total mortality was 31.1% (95%, CI: 24.7–37.5) among individuals with diabetes and kidney disease, which was 11.5% (95%, CI: 7.9–15.2) among people with diabetes but without kidney disease [4]. Moreover, the same patterns were observed for both cardiovascular and non-cardiovascular mortality. More recently, a Taiwan study using a large cohort of half a million adults between 1994 and 2008 found that people with diabetes and early kidney involvement had an average of 16 years of life lost as compared to the general population [5].

Given the adverse health outcomes of DKD, there have been many studies that investigated the effects of the early intervention on such individuals. Glucose-lowering treatment has proven long-term beneficial effects in regards to reducing microvascular complications in type 2 diabetes [6, 7]. The data from the UK Prospective Diabetes Study found that better glycemic control leads to reductions in relative risk of microalbuminuria development and the doubling of plasma creatinine [8]. In addition, a 21-year follow-up study on the Steno-2 randomized trial revealed that compared with conventional treatment, intensified and multifactorial intervention of type 2 diabetes with microalbuminuria for 7.8 years increases median life expectancy by 7.9 years, and that this increase in lifespan is matched by years without incident CVD [9].

The major challenge, however, is early detection and management of the DKD so that intervention can be effective. There is inadequate vigilance to detect early DKD because of less awareness of its seriousness in clinical practice. The survival of people with DKD may differ according to ethnicity, economic status and healthcare system. To our knowledge, there exists no data on the survival outcome of people with type 2 diabetes and DKD in mainland of China. Such information has crucial public health implications because of the current epidemic of type 2 diabetes in China and identifying predictors of excess mortality in people with type 2 diabetes and DKD is important for optimally targeting risk-reduction strategies. In this study, we examined the survival outcome of DKD by levels of estimated GFR (eGFR) and albuminuria in a suburb cohort in Beijing, China and the risk factors for mortality. Life expectancy of individuals with type 2 diabetes and DKD was also calculated.

## Methods

### Data sources

A total of 445 people with type 2 diabetes who was diagnosed as DKD in the Department of Endocrinology of Beijing Pinggu Hospital between January 1st 2003 and December 31st 2013 were enrolled into the study and took the specific questionnaire (Additional file 1). In our study, DKD was defined as persistent albuminuria (≥30 mg/g or ≥ 20 μg/min) that was confirmed on at least two occasions 3–6 months apart without clinical or laboratory evidence of other kidney diseases. The electronic medical records were sources of demographic and clinical information. The participants were followed from inclusion until December 31st, 2015. As of that date, vital status was known for all individuals. Deaths were identified by participant contacts, the Social Security Death Index, or hospital reports, and then confirmed by death certificates. Attempts were made to obtain medical records surrounding the death, autopsy/coroner’s reports, and interview with next of kin regarding the circumstances surrounding the death, as appropriate. Cause of death was classified according to the WHO ICD-10 (www.who.int/classifications/icd/en/) into eight categories: circulatory disease I00-I99; respiratory diseases J00-J99, diabetes-related complications E10-E14, cancers C00-C97, infectious diseases A00-B99, end-stage renal diseases N17-N19, injuries V01-Y98 and others. Diabetes-related complications include diabetes with coma, ketoacidosis, renal complications, peripheral circulatory complications, multiple complications, or unspecified complications.

### Other clinical and demographic measures

The clinical status was evaluated when participants were admitted. Detailed anthropometric measurements were collected by trained nurses, adhering to standardized techniques. Height was measured using a stadiometer, and weight was measured using an electronic scale. The body mass index (BMI) was calculated as weight in kilogram divided by the square of the height in meters. Blood pressure was measured twice with a mercury sphygmomanometer, in the sitting position, and was rounded to the nearest 2 mmHg. The average readings were recorded.

Venous blood samples were taken by nurses after an overnight fast for 8-10 h. Fasting levels of serum creatine, total cholesterol, triglycerides, high-density lipoprotein cholesterol (HDL), and low-density lipoprotein cholesterol (LDL) were measured using an autoanalyzer (Beckman Coulter chemistry analyzer AU5800 series, Miami, FL).

HbA_1c_ concentration was measured by high-pressure liquid chromatography (D-10 Hemoglobin Testing System, Bio-Rad Laboratories, Inc., Schiltigheim, France), and the assay was aligned with the Diabetes Control and Complications Trial. The intra-assay coefficient of variation (CV) for HbA_1c_ was 0.78 and 0.46%, and the inter-assay CVs for HbA_1c_ were 0.52 and 0.53% at mean values of 5.70 and 9.40%, respectively.

Peripheral vascular disease was screened using ultrasonography of lower extremity artery (GE Medical Systems Ultrasound, LOGIQE9, GE Healthcare, Germany). Diabetic retinopathy was screened using non-mydriatic retinal camera (TRC-NW300, TOPCON, Japan) or digital fundus camera (Visucam 224, ZEISS, Germany).

Urine albumin was measured using DCA vantage Analyzer (Siemens Healthcare Diagnostics Inc. NY, USA). eGFR was calculated by the equation of the Chronic Kidney Disease Epidemiology Collaboration [10]. To determine the impact of albuminuria and eGFR on survival, all participants were divided into subgroups according to albuminuria and eGFR obtained from baseline: microalbuminuria group (albuminuria 30–300 mg/d or 20–200 μg/min) and overt albuminuria group (albuminuria > 300 mg/d or > 200 μg/min), and eGFR ≥60 ml/min group and eGFR < 60 ml/min group.

### Statistical analysis

Data were presented as median (25th, 75th Quartile) and mean ± SD or n (%). Demographic and baseline characteristics were compared between groups using Fisher’s exact test for categorical variables and a two-sample Wilcoxon test for continuous variables.

Survival curves were estimated by Kaplan–Meier method and compared by log–rank testing between subgroups with *p* < 0.05 considered as significant. Uni- and multivariate Cox proportional hazard models were applied for risk factor analysis of mortality. In the multivariate model, demographic and baseline characteristics were included as adjustment variables, if the variable showed a significant relationship with mortality in the univariate model. Initial adjustment variables comprised gender, age, BMI, systolic blood pressure (SBP), duration of diabetes, smoke, albuminuria, HbA_1c_ and eGFR.

The proportional hazard assumption was examined and met by time-dependent covariate test. Abridged period life table for those with DKD aged 25 years or older was constructed using the Chiang method by 5-year age intervals up to age 84 years and an open-ended interval thereafter (conditional on surviving to the diagnosis date of DKD) to estimate the remaining life expectancy [11]. CIs were calculated using Monte Carlo simulations [12].

Data analysis was carried out using IBM SPSS Statistics 21.0 (IBM Corporation, NY, USA) and Excel 2007 (Microsoft, Redmond, WA, USA). This study was approved by the ethics committee at Pinggu Campus of Capital University Beijing Friendship Hospital.

## Results

### Participants’ characteristics

The characteristics of this cohort is presented in Table 1. A total of 445 participants with DKD were included in this study, contributing 3232 person-years of follow-up and 78 deaths. Males accounted for 48.8% of this cohort. The mean age at diagnosis of diabetes was 48.8 ± 11.0 years. The median duration of diabetes was 8.0 years (interquartile range [IQR] 4.0–13.0). The mean HbA_1c_ was 82 ± 26 mmol/mol (9.6 ± 2.3%). A total of 59.8% of this cohort had hypertension, among which 70.8% were on treatment of an ACE inhibitor or angiotensin receptor blocker (ARB).

**Table 1:** The baseline characteristics of all study participants

	Total	Microalbuminuria	Overt albuminuria	*P* value
Male gender (n, %)	217 (48.8)	170 (48.4)	47 (50.0)	0.817
Age at registration (years) ^a^	57.5 ± 11.6	57.2 ± 11.6	58.4 ± 11.3	0.397
Age at onset (years) ^a^	48.8 ± 11.0	49.3 ± 10.8	47.1 ± 11.6	0.085
Diabetes duration (years) ^b^	8.0(4.0, 13.0)	7.0 (2.5, 12.0)	10.0 (8.0, 15.0)	0.000
DM family history	192 (43.2)	152 (43.4)	40 (42.6)	0.907
Smoke (n, %)	138 (31.4)	104 (30.1)	34 (36.2)	0.263
Hypertension (n, %)	265 (59.8)	200 (57.1)	65 (69.9)	0.032
BMI (Kg/m^2^) ^a^	26.2 ± 3.8	26.3 ± 3.9	25.8 ± 3.4	0.225
SBP (mmHg) ^b^	130 (120, 145)	130 (120, 140)	140 (120, 151)	0.020
DBP (mmHg) ^b^	80 (80, 90)	80 (80, 90)	80 (80, 90)	0.408
eGFR (ml/min) ^a^	77.1 ± 28.4	64.6 ± 32.3	80.5 ± 26.3	0.000
Total cholesterol (mmol/L) ^a^	4.99 ± 1.31	4.93 ± 1.29	5.21 ± 1.37	0.067
Triglycerides (mmol/L) ^b^	1.72 (1.12, 2.71)	1.70 (1.11, 2.73)	1.86 (1.23, 2.51)	0.563
LDL cholesterol (mmol/L) ^a^	3.05 ± 0.99	3.00 ± 0.95	3.21 ± 1.15	0.109
HDL cholesterol (mmol/L) ^a^	1.10 ± 0.38	1.09 ± 0.39	1.14 ± 0.31	0.334
Uric acid (μmol/L) ^a^	296.4 ± 103.1	286.1 ± 103.3	333.8 ± 93.7	0.000
HbA_1c_ (mmol/mol) ^a^	82 ± 26	83 ± 25	77 ± 27	0.035
FPG (mmol/L) ^a^	10.2 ± 4.0	10.3 ± 4.0	9.5 ± 4.0	0.060
Hypertension (n, %)	265 (59.8)	200 (57.1)	65 (69.9)	0.032
Use of ACE inhibitor/ARB (n, %)	315 (70.8)	242 (68.9)	73 (77.7)	0.125
Diabetic retinopathy (n, %)	108 (27.6)	78 (24.5)	30 (41.1)	0.006
Peripheral vascular disease (n, %)	285 (80.1)	206 (79.5)	79 (81.4)	0.845
Mean age at death (years) ^a^	70.1 ± 9.1	70.6 ± 8.9	68.8 ± 9.7	0.437

^a^Data shown as mean ± SD

^b^Data are medians (IQR)

Participants with overt albuminuria had longer duration of diabetes [10.0 (8.0, 15.0) vs. 7.0 (2.5, 12.0) years, *P* = 0.000], higher levels of SBP [130,140 (120, 151) vs. (120, 140) mmHg, *P* = 0.020] and uric acid (333.8 ± 93.7 vs. 286.1 ± 103.3 μmol/L, *P* = 0.000), lower levels of eGFR (64.6 ± 32.3 vs. 80.5 ± 26.3 ml/min, *P* = 0.000), higher prevalence of hypertension(69.9% vs. 57.1%, *P* = 0.032) and diabetic retinopathy (41.1% vs. 24.5%, *P* = 0.006) but lower level of HbA_1c_ (9.2 ± 2.5% vs. 9.8 ± 2.3%, *P* = 0.035) than those with microalbuminuria at baseline (Table 1).

### Survival rates in participants with type 2 diabetes and DKD by levels of eGFR and albuminuria

**Fig. 1:**
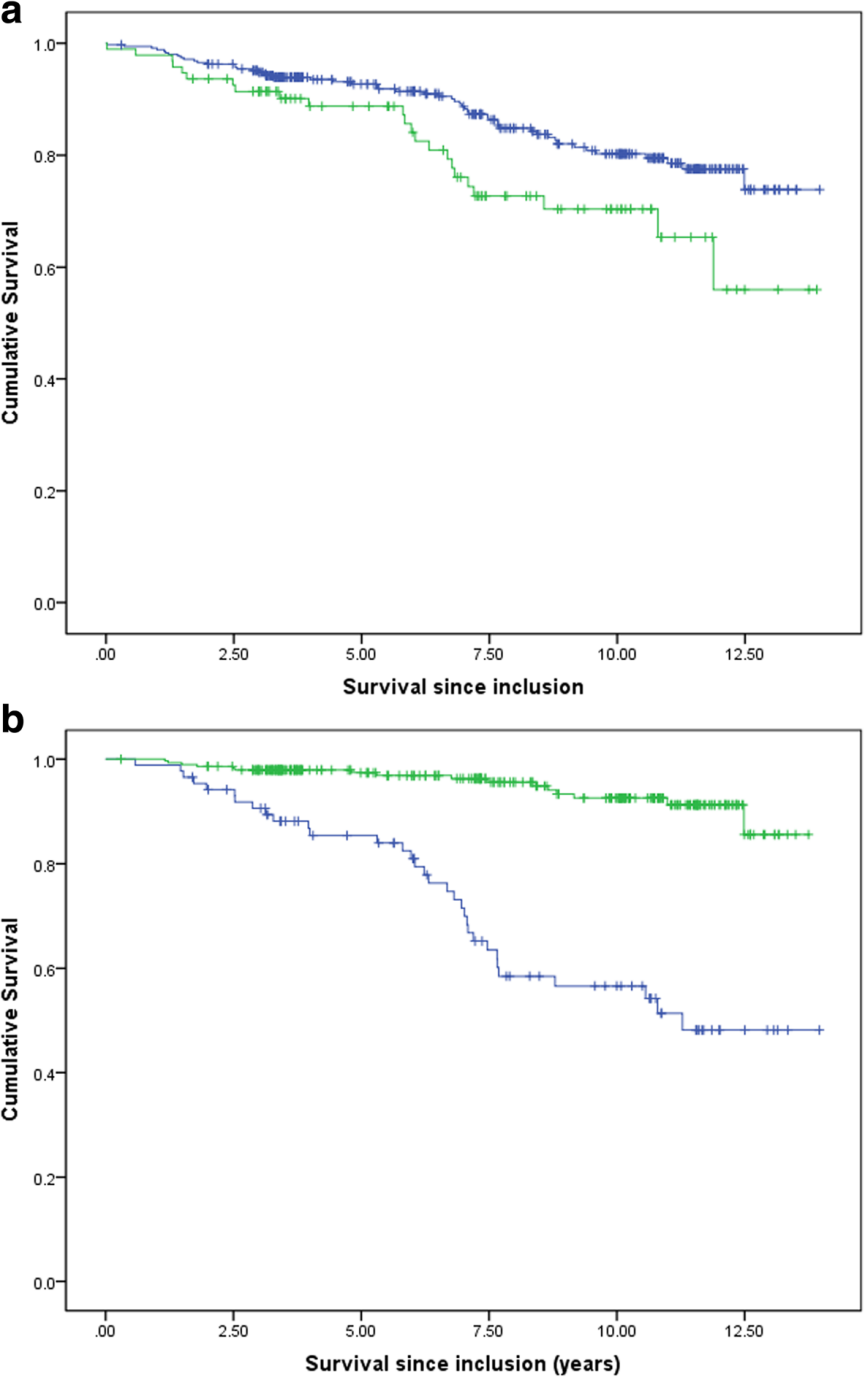
**a** Survival rates of participants with microalbuminuria, compared with those with overt albuminuria. Blue line = microalbuminuria; green line = overt albuminuria. *P* = 0.014 (log rank). **b** Survival rates of participants with eGFR< 60 ml/min, compared with those with eGFR≥60 ml/min. Blue line = eGFR < 60 ml/min; green line = eGFR≥60 ml/min. *P* = 0.000 (log rank)

As shown in the Kaplan-Meier curves in Fig. 1a, crude survival was greater in the microalbuminuria group than in the overt albuminuria group [12.2 (95%, CI: 11.7–12.6) vs.10.9 (95%, CI: 9.9–11.9), *P* = 0.014; log-rank test] (Fig. 1a). The 5- and 10-year cumulative survival rates were 0.927 and 0.802 for the microalbuminuria group, which were 0.888 and 0.703 for the overt albuminuria group.

Crude survival was greater in the eGFR≥60 ml/min group than in the eGFR < 60 ml/min group [12.5 (95%, CI: 12.2–12.9) vs.10.0 (95%, CI: 9.0–10.9), *P* = 0.000; log-rank test] (Fig. 1b). The 5- and 10-year cumulative survival rates were 0.949 and 0.861 for the eGFR≥60 ml/min group, which were 0.810 and 0.587 for the eGFR < 60 ml/min group.

Using the Cox proportional hazard model, as shown in Table 2, the risk of the mortality increased with older age (HR value 1.05, [95%, CI: 1.02–1.09]), higher SBP (1.02, [95%, CI: 1.00–1.03]), lower BMI (0.90, [95%, CI: 0.82–0.98]) and lower eGFR (0.98, [95%, CI: 0.96–0.99]). Life expectancy at an attained age of 50 years was estimated to be an additional 12.3 years (95%, CI: 9.0–16.1) among participants with DKD (Table 3).

**Table 2:** Univariate and Multivariate Cox regression analysis of the risk of mortality in participants with type 2 diabetes and diabetic kidney disease

	Univariate analysis		Multivariate analysis	
	HR (95% CI)	*P* Value	HR (95% CI)	*P* Value
Male gender	1.11 (0.71–1.75)	0.648		
Age at registration	1.08 (1.05–1.10)	0.000	1.05 (1.02–1.09)	0.003
Smoke	0.76 (0.45–1.28)	0.302		
Diabetes duration	1.03 (0.99, 1.07)	0.074	0.97 (0.92–1.01)	0.163
BMI	0.89 (0.82, 0.97)	0.006	0.90 (0.82–0.98)	0.017
SBP	1.02 (1.01–1.03)	0.002	1.02 (1.00–1.03)	0.010
eGFR	0.97 (0.96–0.98)	0.000	0.98 (0.96–0.99)	0.001
HbA_1c_	1.04 (0.94, 1.15)	0.438	1.12 (0.98–1.28)	0.108
Overt albuminuria	1.83 (1.12, 2.99)	0.016	0.82 (0.41–1.63)	0.569

**Table 3:** Abridged Period Life Table for participants with type 2 diabetes diabetic kidney disease

Age	Observed deaths	Death Rate (per 1000 PY)	Estimated Remaining Life Expectancy, (95% CI), years
25–29	0	0	37.3 (34.1, 41.5)
30–34	0	0	32.3 (29.2, 36.5)
35–39	0	0	27.3 (24.1, 30.1)
40–44	0	0	22.3 (19.0, 26.3)
45–49	0	0	17.3 (14.1, 21.4)
50–54	5	70.4	12.3 (9.0, 16.1)
55–59	7	57.4	11.5 (9.1, 14.5)
60–64	12	98.4	9.6 (7.4, 12.2)
65–69	16	99.4	9.1 (7.3, 11.5)
70–74	12	74.5	8.5 (6.8, 10.7)
75–79	11	144.7	6.3 (4.6, 8.3)
80–84	12	157.9	5.6 (4.3, 7.3)
85+	3	214.3	4.7

### Causes of death in participants with type 2 diabetes and DKD

Overall, circulatory disease was the leading cause of death in participants with type 2 diabetes and DKD (accounting for 43.6% of all deaths), followed by diabetes-related complications (accounting for 33.3% of all deaths) (Table 4). The mean age at death was 70.1 ± 9.1 years, and the median duration of diabetes at death was 10.0 years (IQR 4.8–14.3) (Table 5).

**Table 4:** The major causes of death in the cohort

Cause of death	n (%)
Circulatory disease	34 (43.6)
Respiratory disease	5 (6.4)
Diabetes related complication	26 (33.3)
Cancer	4 (5.1)
Infections	4 (5.1)
ERSD	1 (1.3)
Injury	1 (1.3)
Others	3 (3.8)
Total	78 (100)

**Table 5:** The characteristics of all deaths in the cohort

Male gender (n, %)	Age at onset (years) ^a^	Diabetes duration (years) ^b^	Age at death (years) ^a^	BMI (Kg/m^2^) ^a^	HbA_1c_ (mmol/mol) ^a^
36 (46.2)	55.2 ± 11.2	10.0 (4.8, 14.3)	70.1 ± 9.1	24.7 ± 3.2	84 ± 30

^a^Data shown as mean ± SD

^b^Data are medians (IQR)

## Discussion

In this Chinese cohort, we found that people with type 2 diabetes and DKD in our study faced higher risks of death and shorter life expectancy. This is the first report on survival of people with type 2 diabetes and DKD in mainland of China. The estimated life expectancy at birth for the general Chinese population in 2010 (the midpoint year for our cohort) was ~ 74.8 years. The life expectancy at an attained age of 50 was estimated to be 12.3 years in this cohort, while a study from Taiwan reported that life expectancy at 50 years old for people with type 2 diabetes and early kidney involvement was about 25 years [5]. The potential contributors to the great life loss in our cohort include the limited recourses to manage diabetes and insufficient control of hypertension and dyslipidemia to reduce the risk of cardiovascular disease. First, glycemic control is not optimal in our cohort, which had a mean baseline glycated HbA_1c_ level of greater than 9.5%. The 3B study in 2013 which included a nationally representative sample of the diabetic population in China, reported that the mean HbA_1c_ level was 7.6% [13]. A Denmark study including all people with type 2 diabetes and overt albuminuria at the Steno Diabetes Center showed that the mean HbA_1c_ level was 8.6% [14]. The data from these two studies suggest that the control of blood glucose in our cohort was far worse. Second, nearly 60% of participants in the current study had comorbid hypertension; this proportion is comparable to findings from the Taiwan study, which also included people with diabetes and early kidney involvement [5]. For people with diabetes and albuminuria, inhibition of the RAS is recommended because mounting evidence have suggested its benefits on delaying onset of the combined end point of doubling of serum creatinine, end stage renal disease, or mortality [15–17]. However, in our study, the treatment of RAS inhibition were used only in 70% of participants, which reached over 90% in Denmark cohort [14]. In addition, the mean total serum cholesterol was 5.0 mmol/L in our study, and only 38.9 and 35.7% of participants achieved adequate control of blood lipid for total serum cholesterol and LDL-c, respectively (the recommended target goals of total serum cholesterol was set as < 4.5 mmol/L and LDL-c as < 2.6 mmol/L by reference to the Chinese guidelines for diabetes prevention and treatment). Thus, achieving adequate control of modifiable cardiovascular risk factors in people with type 2 diabetes and DKD faces a range of challenges.

In our study, participants with poor kidney function which is evaluated by albuminuria or eGFR had significantly poorer 5- and 10-year survival rates. Using the Cox proportional hazard model, we found that older age, lower BMI, higher SBP and lower eGFR were associated with higher risk for mortality. In 2012, a meta-analysis that included over 1 million participants reported that higher urinary albumin creatinine ratio and lower eGFR increased the risk of all-cause and cardiovascular mortality in individuals with or without diabetes, but the absolute risks of all-cause and cardiovascular mortality were 1.2–1.9 times higher in patients with diabetes than in those without diabetes throughout the entire ranges of urinary albumin creatinine ratio and eGFR [3].

In addition, our data suggest that lower BMI was associated with poor survival among participants with type 2 diabetes and DKD. Many studies have documented an inverse association between BMI and total mortality in people with type 2 diabetes, an effect called the “obesity paradox”. This paradox generally does not fit more severe degrees of obesity, wherein most studies show poor prognosis with BMI >35Kg/m^2^. A U-shaped relationship between total mortality and BMI was reported with the minimum mortality around BMIs 22.5–25 Kg/m^2^ in a longitudinal cohort study including 2161 Taiwanese with type 2 diabetes [18]. Potential mechanisms underlying this paradox may be the protective effect of obesity against frailty and wasting diseases, more severe diabetes among normal-weight persons with diabetes, or the effect of a “metabolically obese normal weight” phenotype [19].

Blood pressure is a well established cause of vascular disease. Positive associations between SBP and risk of major vascular disease were reported in a nationwide prospective study in China, with an association between each 10 mmHg increase of usual SBP and 36% higher risk of major vascular disease [20]. Another Indian study indicated that per 20 mmHg increase in usual SBP was associated with 2.5-fold higher risk of stroke mortality, 1.7-fold higher risk of cardiac mortality and 1.8-fold higher risk of all vascular mortality [21]. Consistent with the results of previous analyses, findings in our cohort showed that higher SBP is related to higher risk of death.

An analysis of the cause of death in our study showed CVD as the most common cause in over 40% of cases, followed by diabetes related complications which comprised 33% of the causes of death. Several studies have found that mortality due to CVD has declined over recent decades for people with or without diabetes, but it is still one of the leading causes of death in type 2 diabetes [22]. Cause of death was determind by using death certificate, which is a relatively unreliable source of information [23, 24]. Harding et al. found that the proportion of deaths due to CVD underestimated by 26% among type 2 diabetes. With an increasing awareness of the role of diabetes, the underlying cause of death may be given as diabetes related complications when the death was primarily caused by CVD. This may occur in our study and lead to misclassification and an underestimate of the impact of CVD.

Our study has certain limitations and the results must be interpreted with caution. First, our study comprised hospital-based data and the cohort was from one of suburbs in Beijing with relatively poor glycaemic control and low prevalence of ACEI/ARB use, which may limit the generalizability of the findings and lead to overestimation of the excess mortality in people with DKD. Second, relatively few participants had advanced DKD with eGFR< 30 ml/min. So, the results of our study may underestimate the excess mortality in people with DKD in mainland of China. Third, sample size was small which might reduce the power of the study and increase the margin of error. When constructing life table, large sample size can help to obtain close 95% CIs, especially among younger age intervals where fewer deaths occur. In our study no death occurred under the age of 45 years old, which may cause underestimation of the excess mortality. Fourth, some of the factors influencing mortality, such as cardio and cerebral vascular disease, were not available. The survival status might differ considerably for certain patients groups. However, the risk factors of CVD have been adjusted in the analysis. In addition, there could be some nondifferential misclassification bias of baseline clinical characteristics, and we have not accounted for changes in blood pressure, HbA1c, albuminuria and other risk factors over time. Finally, albuminuria is a marker for kidney disease or kidney damage [25]. But not all people with DKD and reduced eGFR have increased albuminuria [8]. Our study did not capture those with DKD who present with reduced eGFR but normal albuminuria. Moreover, albuminuria is measured by a urine sample at each visit or admission, and day to day variability may reduce the precision of the results.

## Conclusions

Data from this cohort in Beijing from 2003 through 2015 showed that people with type 2 diabetes and DKD faced higher risk of death and shorter life expectancy. Factors significantly increasing risk of death included older age, higher SBP, lower BMI and lower eGFR. The increasing burden of diabetes and poor survival rates of people with DKD together imply that there is an urgent need to implement large-scale screening and awareness programs on DKD in China. Once DKD is detected, closely monitoring and multifactorial intervention should be executed.

## Supplementary information

**Additional file 1.** Questionnaire of economic cost and survival status of diabetic nephropathy in Pinggu area.

## Data Availability

The datasets used and/or analysed during the current study are available from the corresponding author on reasonable request.

## Abbreviations

ARB: Angiotensin receptor blocker
BMI: Body mass index
Ccr: Creatinine clearance rate
CV: Coefficient of variation
CVD: Cardiovascular disease
DKD: Diabetic kidney disease
GFR: Glomerular filtration rate
HDL-c: High-density lipoprotein cholesterol
IQR: Interquartile range
LDL-c: Low-density lipoprotein cholesterol
SBP: Systolic blood pressure
