# Supplementary material for: Survival of Chinese people with type 2 diabetes and diabetic kidney disease: a cohort of 12 -year follow-up
Source: BMC Public Health. 2019 Nov 9;19:1498. doi: 10.1186/s12889-019-7859-x (PMC6842464; doi:10.1186/s12889-019-7859-x)
Supplement: Supplementary file 1 — Additional file 1. Questionnaire of economic cost and survival status of diabetic nephropathy in Pinggu area. [file 12889_2019_7859_MOESM1_ESM.docx]

**Economic cost and survival status of diabetic nephropathy in**

**Pinggu area**

**Questionnaire**

**Beijing Pinggu Hospital**

**2015**

We will keep all your information confidential, please fill in according to your actual situation. (Please fill in the answer number on ________)

Name：________________

Chinese citizenship ID ：________________________________

Home address ：________________________________

Respondent's personal number：___________

Contact：Home phone number 1：___________ Home phone number 2：__________

Telephone 1：_______________ Telephone 2：______________

**Part 1: Demographic information**

1、Name（Full name）：___________

2、Gender：（1）male （2）female

3、Date of birth ：________________

4、Age ： ______

5、Nationality： （1）Han （2）other ________

6、Highest education completed： ________

(1) Not receiving formal school education (2) Elementary school and below (3) Graduation from junior high school (4) Graduation from high school/secondary school (5) Graduation from college/college (6) Graduate graduates and above

7、Current marital status: ________

(1) Single (2) Separated (3) Widowed (4) Divorced (5) Cohabitation

(6) Married

8、Current occupation: ________

(1) = workers (2) = farmers (3) = technology (4) = administration

(5) =teacher (6) =finance or business (7) = medical services

(8) =driver (9) =Housework (10) =Student

(11) = retired (12) =Other

9、Your monthly income: ________

(1) = “<RMB 500 yuan” (2) = “RMB 500-999 yuan” (3) = “RMB 1000-1999 yuan” (4) = “RMB 2000-2999 yuan” (5) = “RMB 3000-3999 yuan”

(6) = “RMB 4000-4999 yuan” (7) = “> RMB 5000 yuan”

10、The number of permanent residents of your family: ________ people

11、Total income of your family in the past 1 year: ________

(1) "< 5000 yuan" (2) = "5000 yuan - 9999 yuan"

(3) = "10,000 - <2.5 million" (4) = "2.5 million - <5 million"

(5) = "50,000 - <7.5 million" (6) = "7.5 million - <100,000"

(7) = "≥ 100,000 yuan" (8) = " unknown "

**Part II: Health and Medication History**

1. The first time when you were diagnosed as having diabetes? ____ (mm/yyyy)

2. type of diabetes : ________

(1) Type 1 (2) Type 2 (3) Other types (4) unknown

3. Do you have a family history of diabetes? ________

(1) Yes, if any, please specific to the person _______________

(2) None (3) unknown

4. Do you have a family history of cancer? ________

(1) Yes, if any, please specific to people and diseases ________________

(2) None (3) unknown

5. Were you diagnosed as having chronic complications of diabetes? ________

(1) None (2) Hypertension (3) Coronary heart disease (4) Hyperlipidemia (5) Stroke (6) Fatty liver

(7) Tumor (if any): ___________

(8) Others (if any): ___________

(9) Unknown

6. If there are other chronic diseases, which hospital is diagnosed? ______________

7. Have you been told by a doctor about chronic complications of diabetes?

| (1) Diabetic retinopathy 0=No 1=Yes |
| --- |
| (2) Diabetic nephropathy 0=No 1=Yes |
| (3) Diabetic neuropathy 0=No 1=Yes |
| (4) Lower extremity atherosclerosis 0=No 1=Yes |
| (5) Diabetic foot 0=No 1=Yes |
| (6) Others 0=No 1=Yes, Name________________ |
| please specify the name of the medicine:___________________________________ |

8. Have you ever had hypoglycemia? Answer 2 skip to 12） __________

(1) Yes (2) No (3) unknown

9. How often do you have hypoglycemia: ________

(1) Appears every day (2) 1-2 times/week (3) 1-2 times/month

(4) Less than 1-2 times a year (5) no regularity

10．The lowest value you can detect when you have hypoglycemia _______mmol / L.

11. What are the symptoms of your hypoglycemia? (Multiple choice)________

(1) Palpitations and sweating (2) dizziness, blurred vision

(3) Unconsciousness (4) Nausea, fatigue (5) other ________________

12. When was the last time you tested blood glucose, blood lipids and other biochemical indicators? ________

(1) = "within 1 month" (2) = "2-3 months" (3) = "4-6 months"

(4) = "7-12 months" (5) = "more than 12 months"

(6) = "Do not check" (7) = “unknown”

13. When was the last time you tested glycosylated hemoglobin? ________

(1) = "within 1 month" (2) = "2-3 months" (3) = "4-6 months"

(4) = "7-12 months" (5) = "more than 12 months"

(6) = "Do not check" (7) = “unknown”

14. When was the last time you tested your blood routine? ________

(1) = "within 1 month" (2) = "2-3 months" (3) = "4-6 months"

(4) = "7-12 months" (5) = "more than 12 months"

(6) = "Do not check" (7) = “unknown”

15. When was the last time you tested your urine routine? ________

(1) = "within 1 month" (2) = "2-3 months" (3) = "4-6 months"

(4) = "within 7-12 months" (5) = "more than 12 months"

(6) = "Do not check" (7) = “unknown”

16. When was the last time you tested urine protein? (Including urine protein creatinine ratio, urinary protein excretion rate, 24-hour urine protein) ________

(1) = "within 1 month" (2) = "2-3 months" (3) = "4-6 months"

(4) = "within 7-12 months" (5) = "more than 12 months"

(6) = "Do not check" (7) = “unknown”

17. How do you monitor finger blood sugar? (Multiple choice)________

(1) Hospital (2) Pharmacy (3) Self-prepared blood glucose meter

(4) No monitoring

18. How often do you monitor the blood glucose level? ________

(1) Every day (2) 3-5 times/week (3) 1-2 times/month

(4) less than 1-2 times per year (5) no monitoring (6) Irregular

19. Do you consult a doctor after detecting abnormal blood glucose? ________

(1) every time (2) sometimes, as the case may be (3) Never consult

20. When did you diagnose diabetic nephropathy: ____ (mm/yyyy)

21. In which medical institution is diagnosed with diabetic nephropathy? _______

(1) = hospital examination (2) = regular physical examination (3) = crowd screening (4) = unknown

22. What was the level of the medical facility where you were diagnosed diabetic nephropathy? ________

（1）=Level 3 （2）= Level 2

（3）= Level 1 （4）=other

（5）=unknown , please specify _______________

23. Which of the following diabetic nephropathy symptoms do you have when you first diagnose diabetic nephropathy? (Multiple choice)________

(1) Anemia (2) edema (3) hypertension

(4) Pleural effusion (5) fatigue (6) asymptomatic

(7) Other ___________

24. What are the hazards of diabetes nephropathy? (Multiple choice)________

(1) Anemia (2) edema (3) hypertension

(4) Pleural effusion (5) weight loss and fatigue (6) hemodialysis

(7) life-threatening

(8) Other _____________

(9) Unknown

25. Are there diabetic nephropathy among relatives who are related to you? ________

(1) If yes, please be specific to: _____________

(2) No (3) Unknown

26. What measures do you take to control your blood sugar? (Multiple choice) _______

(1) Oral hypoglycemic agents according to doctor's advice (2) Control diet (3) Moderate exercise (4) Insulin (5) No measures

(6) Other _________

27.Are you currently taking any of the following anti-diabetic medications?

| （1）Biguanide 0=no 1=yes |
| --- |
| （2）Sulphonylurea 0=no 1=yes |
| （3）Glinides 0=no 1=yes |
| （4）glycosidase inhibitors 0=no 1=yes |
| （5）Thiazolidinediones 0=no 1=yes |
| （6）DPP-4 0=no 1=yes |
| （7）other 0=no 1=yes，please specify ________________  Cannot determine the name of the drug that can be written：__________________  ___________________________________________________________________ |

28. Do you use insulin?？（1）yes （2）no ________

If you apply insulin, please indicate how long it has been applied so far?  **______**(mm/yyyy)，choose “No” please skip to question 29.

| What is your daily dose of insulin ______ unit(s) |
| --- |
| （1） Short-acting insulin 0=no 1=Yes |
| （2）human intermediate insulin 0=no 1=yes |
| （3）human premixed insulin 0=no 1=yes |
| （4）long acting insulin analogue 0=no 1=yes |
| （5）rapid acting insulin analogue 0=no 1=yes |
| （6）premixed insulin analogue 0=no 1=yes |
| （7）Animal short-acting insulin 0=no 1=Yes |
|  |
| f you can't judge the category, please fill in the common name of the specific drug on the horizontal line:  The name of insulin currently used: _________________________________________ |

29. Do you control blood sugar according to your doctor's advice? ________

(1) Completely (2) Increase or decrease of dose by yourself

(3) Occasionally forgotten (4) always forget to take the medicine

(5) Change the medicine by yourself (6) Stop the medicine by yourself

30. The drug you are currently using (please specify the specific drug name)

| Drug type | 0=No  1=Yes  9=Unknown | Drug type | 0=No  1=Yes  9=Unknown | Drug type | 0=No  1=Yes  9=Unknown |
| --- | --- | --- | --- | --- | --- |
| ACEI |  | Angiotensin receptor antagonist |  | Calcium antagonist |  |
| β blocker |  | Diuretics |  | Aspirin |  |
| Statins |  | Bate lipid-lowering drug |  | Nicotinic acids |  |
| Nitrate drug |  | Glucocorticoids (in the past year) |  | Thyroid hormone (in the past year) |  |
| Folic acid |  | Iron agent |  | Sodium bicarbonate |  |
| KaiTong |  | BaiLing |  | JinShuiBao |  |
| If you can't classify, please write down all current medications：__________________________________________________________________________ | | | | | |

31. Have you applied antibiotics in the past 1 month? ________

(1) Yes, if any, please indicate __________________________________

(2) No

32. Is erythropoietin currently used? ________

(1) Yes (2) No (please skip to 35 questions)

33. Please indicate the time of application of erythropoietin: ___ (mm/yyyy)

34. Please indicate the name and frequency of administration of erythropoietin.

Application of erythropoietin name: ______________

Frequency of medication: _____________

35. Do you dialysis treatment now? ________

(1) Yes, if dialysis is performed, please specify the specific time ___ (mm/yyyy)

(2) No

36、Your current monthly total medical expenses：________

(1) = “<RMB 500 yuan” (2) = “RMB 500-999 yuan” (3) = “RMB 1000-1999 yuan” (4) = “RMB 2000-2999 yuan” (5) = “RMB 3000-3999 yuan”

(6) = “RMB 4000-4999 yuan” (7) = “> RMB 5000 yuan” (8) = “Unknown”

37. The total medical expenses you need to pay per month except for medical insurance ________

(1) "< 500 yuan" (2) = "500-999 yuan" (3) = "1000-1999 yuan"

(4) = "2000-2999 yuan" (5) = "3000-3999 yuan" (6) = "4000-4999 yuan"

(7) = "≥ 5000 yuan"

38. What do you think is the most troublesome problem for you? (Multiple choice) ________

(1) Economic expenses (2) Quality of life (3) Concerns about the future

(4) Discrimination (5) No problem (6) others

**Part III Living habits**

1、Do you currently drink alcohol? (Refers to an average of two or more drinks per week for more than one year, the choice of drinking but below this standard "occasionally") ________

(1) No (2) Occasionally (3) Yes, now (4) drank in the past, but has quit. (More than 1 year)

[Select (1) jump to 6, choose (3) skip 5 alcohols]

2、If yes, at what age did you start to drink? _____ years old

3、How many years have you been drinking? ______year

4、The type and amount of drinking: [not drinking without filling]

(1) Liquor (degrees are: ) times ∕______ (time) ml/times

(2) Beer (degrees are: ) times ∕______ (time) ml/times

(3) wine (degrees are: ) times ∕______ (time) ml/times

(4) Medicinal liquor: times / ______ (time) ml/times

5、Stop drinking

(1) Have you stopped drinking? (1) Yes (2) No

(2) How many years have you stopped drinking? ______year

(3) Reasons of stop drinking: ① sick ② economic reasons

③family opposition ④ recognize the danger of drinking

⑤other: ______________________

6、Do you smoke cigarettes now (or ever)? (1) Yes (2) No [Select (1) Jump to 11]

7、If you smoke at present, how many cigarettes per day on average do you

Smoke? ____ (1) Occasionally (2) 1-5 (3) 6-10 (4) more than 10

8、How many years have you been smoking? : ______ (mm/yyyy)

9、Do you stop smoking now? ________

(1) Yes (2) No

10、How many years have you quit smoking: ______ (mm/yyyy)

11、Do you exercise? ________

(1) No exercise (2) Less than 30 minutes per day (3) More than 30 minutes per day (4) less than 30 minutes per week

12.、The main ways of normal exercise: (1) Walking (2) running

(3) Other ____________

13、your actual sleep time ________ hours per day

14. Do you feel that your work or life is under great pressure? ________

(1) never (2) rarely (3) sometimes (4) often (5) always

**Part IV Relevant disease cognition level**

| 1. Do you think you need to check your urine protein regularly after suffering from diabetic nephropathy? 0=no 1=yes 9=unknown |
| --- |
| 1. Do you think you need to control blood pressure and blood lipids after suffering from diabetic nephropathy? 0=no 1=yes 9=unknown |
| 1. Do you know your staging of diabetic nephropathy? 0=no 1=yes |
| 1. Do you think that diabetic nephropathy needs to control soy products?   0=no 1=yes 9=unknown |

**Part V: Food Frequency Questionnaire**

1. Eating habits
2. Your diet is composed of: _____ (If 4: skip to Question 2)

1=mainly meat 2=half meat, half veggie 3=mainly vegetarian 4=completely vegetarian

1a. what kind of meat do you primarily eat? ____
 1=mostly white meat 2=mostly red meat

3=half red meat and half white meat

1b. what could you consider your diet’s main meat consistency to be? ____
1=lean 2=half lean, half fatty 3=fatty

1c. What do you eat when you eat eggs? _______

1=protein 2=egg yolk 3=whole egg 4=do not eat eggs

2. Your diet’s saltiness is: _____

1=not salty 2=regular saltiness 3=slightly salty 4=very salty

1. How often do you eat snacks? ( Purchased snacks, not home-made )____ （If you choose 0, skip to 5 questions.）

0=Do not eat 1=rarely (<1-3 times/month) 2=Sometimes (1-2 times/week) 3=frequently (3-6 times/week) 4=very often (every day)

4. How often do you eat dessert items (cakes, candies, pastries, other sweet treats)? ________

0=Do not eat 1=Rarely (<1-3 times/month) 2=Sometimes (1-2 times/week) 3=frequently (3-6 times/week) 4=very often (every day)

5. How often do you eat fried foods? ：______

0=Do not eat 1=Rarely (<1-3 times/month) 2=Sometimes (1-2 times/week) 3=frequently (3-6 times/week) 4=very often (every day)

6. How often do you eat [coarse food grains](https://fanyi.sogou.com/?keyword=coarse%20food%20grains&fr=websearch_submit&from=en&to=zh-CHS)? ：______

0=Do not eat 1=Rarely (<1-3 times/month) 2=Sometimes (1-2 times/week) 3=frequently (3-6 times/week) 4=very often (every day)

| **Interviewer** |  |
| --- | --- |
| \| 1. How did the participant cooperate with you during the interview? _____   1=very well 2=well 3=OK 4=poorly \|  \| \| --- \| --- \| \| 1. In general, how do you rate the quality of this survey: _____   1=reliable 2=almost reliable 3=not reliable  3. Please confirm the date of this survey （yyyy-mm-dd)  4. Signature of interviewer :_____________________________  5. Quality controller signature: _____________________________ \|  \| |  |

**Part VI Physical examination**

1. Blood Pressure：

First reading： / mmHg；

Second reading： / mmHg；

Third reading： / mmHg；

Was the blood pressure taken while the participant sits calmly for at least 10 minutes, feet flat on floor? 0=no 1=yes 9=unknown

If the answer is “0” to the question above, explain the reason why the protocol was modified:

__________________________________________________________________

1. Height: _____ cm
2. Weight: _____ kg

4. Waist (Flat umbilical hole): feet apart 30-40cm，With a tape measure at the level of the umbilicus, measure circumference level，tape measure should be close to the skin, but not oppressive (accurate to 0.5cm).

. cm

5. Hip: _____ cm

Physical examination signature:：

**Part VII：Electrocardiograph**

Result ：

**Part VIII：Fundus examination**

Left：

Right：

**Part IX：Neuropathy Physical Assessment**（Limb arterial examination plus measurement and low intermediate frequency electrical diagnosis）

Left ：

Right ：

**Part 10：Laboratory Tests**

1. fasting plasma glucose **___** mmol/l
2. HbA1c **__** %
3. total cholesterol mmol/l
4. triglyceride mmol/l
5. LDL-C mmol/l
6. HDL-C mmol/l
7. serum ALT U/L
8. serum AST U/L
9. TP g/L
10. ALB g/L
11. TBIL umol/l
12. TBA umol/l
13. BUN mmol/l
14. serum creatinine umol/L
15. serum uric acid umol/L
16. serum K **___** mmol/l
17. serum NA **___** mmol/l
18. serum CL **___** mmol/l
19. HS-CRP mg/l
20. FT4 pmol/l
21. TSH uIU/ml
22. TPO U/ml
23. White blood cell count × 10^9^/L
24. Red blood cell count × 10^12^/L
25. hemoglobin g/l
26. Blood platelet count × 10^9^/L
27. HCT %
28. MCV fl
29. MCH pg
30. MCHC g/l
31. RDW-SD %
32. stool routine：
33. Urine routine：
34. A/C(urine protein creatinine ratio)
35. （Discretion）24-hour urine protein quantitation g/l

Laboratory data filler signature：

**Questionnaire instruction:**

1. A questionnaire has been signed for informed consent.

2. The questionnaire needs to be filled out by the investigator with a blue-black or black pen.

3. The questionnaire must be filled in accurately and cannot be altered. When correcting the error, you need to draw the horizontal line and sign the modifier's initials and modification time. Do not cover up the original data.

4. All items on each page of the questionnaire should be filled in.

5. Cannot provide, not applicable, or unknown to be represented by the number "999".

6. Medical knowledge:

Resident population: refers to people who live together for a long time

Red meat: red meat before cooking. All mammalian meat is red meat.

White meat: broad meaning, including birds, fish, reptiles, amphibians, crustaceans.

Coarse grains: corn, millet, purple rice, sorghum, oats, buckwheat, wheat bran and various dried beans in cereals, such as soybeans, green beans, red beans, mung beans, etc. and tubers. Refers to food other than rice and flour.

Occasional smoking: less than one daily average.

The degree of wine: low alcohol below 20 degrees, high alcohol above 40 degrees, and moderate wine at 20-40 degrees. Writable range.

The number of milliliters of wine: the bottle is generally 550-600ml, and the can is 330-500ml.

Erythropoietin: EPO for short, also known as recombinant human erythropoietin for injection. Product name: Ebion.

Diabetes complications:

large blood vessels: coronary heart disease, cerebral infarction, cerebral hemorrhage, lower extremity atherosclerosis, carotid atherosclerosis.

Microvessels: retinopathy, peripheral neuropathy, autonomic neuropathy, kidney disease

Short-acting insulin: Novo Ling R\Yumulin R\ Gan Shulin R

Moderate Insulin: Novo Ling N\Yumulin N\ Gan Shulin N

Premixed insulin: Novo Ling 30R\Nuoheling 50R\Youlin 30R\Youlin 50R

Long-acting insulin analogues: insulin glargine, insulin detemir

Fast-acting insulin: Novo

Premixed analogues: Novo and Sharp 30/50, Uber 25

7. If the confirmed patient in Pinggu District Hospital is diagnosed before 2014, the level is level 2, and if it is later, the level is level 3.

8. The name of the drug in question 27, if it is clear "yes", mark "√", if it is clear "No" mark"√", whether it is unclear whether to write the name of the drug, do not make no judgment.
